# A rare case report of epithelioid hemangioendothelioma involving both lungs and right atrium with multi-system involvement

**DOI:** 10.3389/fmed.2026.1897921

**Published:** 2026-09-10

**Authors:** Qingqing Shao, Zhenkui Chen, LiYa Zhao, Tao Li, Xudong Ai

**Affiliations:** 1 Department of Pathology, The Fifth Affiliated Hospital of Dali University, Baoshan, Yunnan Province, China; 2 Department of Ultrasound, The Second Hospital of Baoshan, Baoshan, Yunnan Province, China; 3 Department of Radiology, The Fifth Affiliated Hospital of Dali University, Baoshan, Yunnan Province, China; 4 Radiological Department, Tongji Hospital, Tongji Medical College, HUST, Wuhan, China

**Keywords:** both lungs, case report, epithelioid hemangioendothelioma, multi-system involvement, PET-CT/MRI, right atrium

## Abstract

Epithelioid hemangioendothelioma (EHE) is a rare low-grade malignant vascular neoplasm characterized by local aggressiveness and metastatic potential. Clinically, EHE may involve multiple anatomical sites—including the lungs, liver, bones, and soft tissues—either synchronously or metachronously. Its non-specific clinical manifestations often complicate early diagnosis. Given the extreme rarity of documented cases in the literature, we report a rare case of right atrial EHE with concurrent bilateral pulmonary and extensive systemic metastases. Furthermore, we present a comprehensive literature review to explore the diagnostic and therapeutic challenges associated with this disease. We particularly emphasize the necessity of a thorough diagnostic evaluation encompassing both pathological and imaging modalities to ensure accurate characterization and diagnosis of the tumor in this case.

## Introduction

Epithelioid hemangioendothelioma (EHE) represents an exceedingly rare vascular neoplasm of low-grade malignancy, characterized by angiogenic proliferation, local tissue invasiveness, and metastatic potential (12). Clinically, EHE has a propensity for multi-organ involvement, with synchronous or metachronous dissemination to the lungs, liver, bones, and soft tissues being well documented, though its manifestations often lack pathognomonic features, leading to diagnostic delays (6, 7, 9, 11). Despite advancements in imaging and molecular pathology, the rarity of EHE—with fewer than one case per million population reported annually—has limited large-scale clinical studies, resulting in a paucity of standardized treatment protocols (4).

Cardiac involvement by EHE is exceptionally uncommon, accounting for less than 5% of all cases, with right atrial localization and systemic metastases representing an even more extraordinary phenomenon (5). Herein, we describe a rare case of primary right atrial EHE complicated by bilateral pulmonary and extensive osseous/soft-tissue metastases, highlighting the diagnostic challenges posed by its non-specific clinical presentation and radiological mimicry of other vascular tumors. In addition, we provide a comprehensive review of the literature to elucidate the epidemiological, histopathological, and therapeutic nuances of this enigmatic entity.

## Case reports

**Figure 1:**
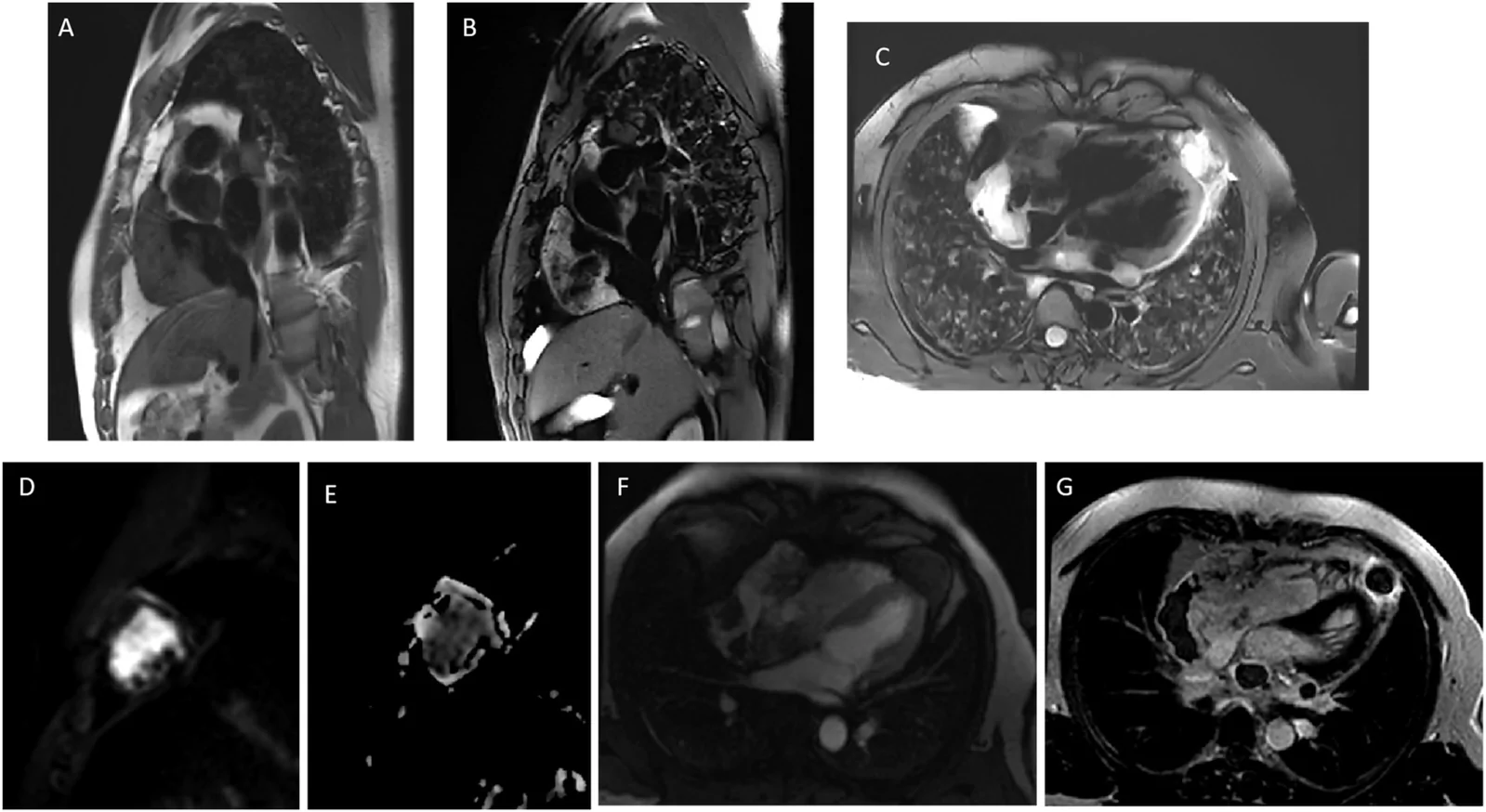
MRI (cardiac plain + function + perfusion + delayed enhancement): **(A,B,C,F,G)** show multiple soft-tissue nodules/masses around both atria and apex, right atrial involvement, increased mediastinal/epicardial fat, and minimal pericardial fluid. Diffuse lung nodules/patches are visible. **(D,E)** indicate marked diffusion restriction of lesions on DWI.

**Figure 2:**
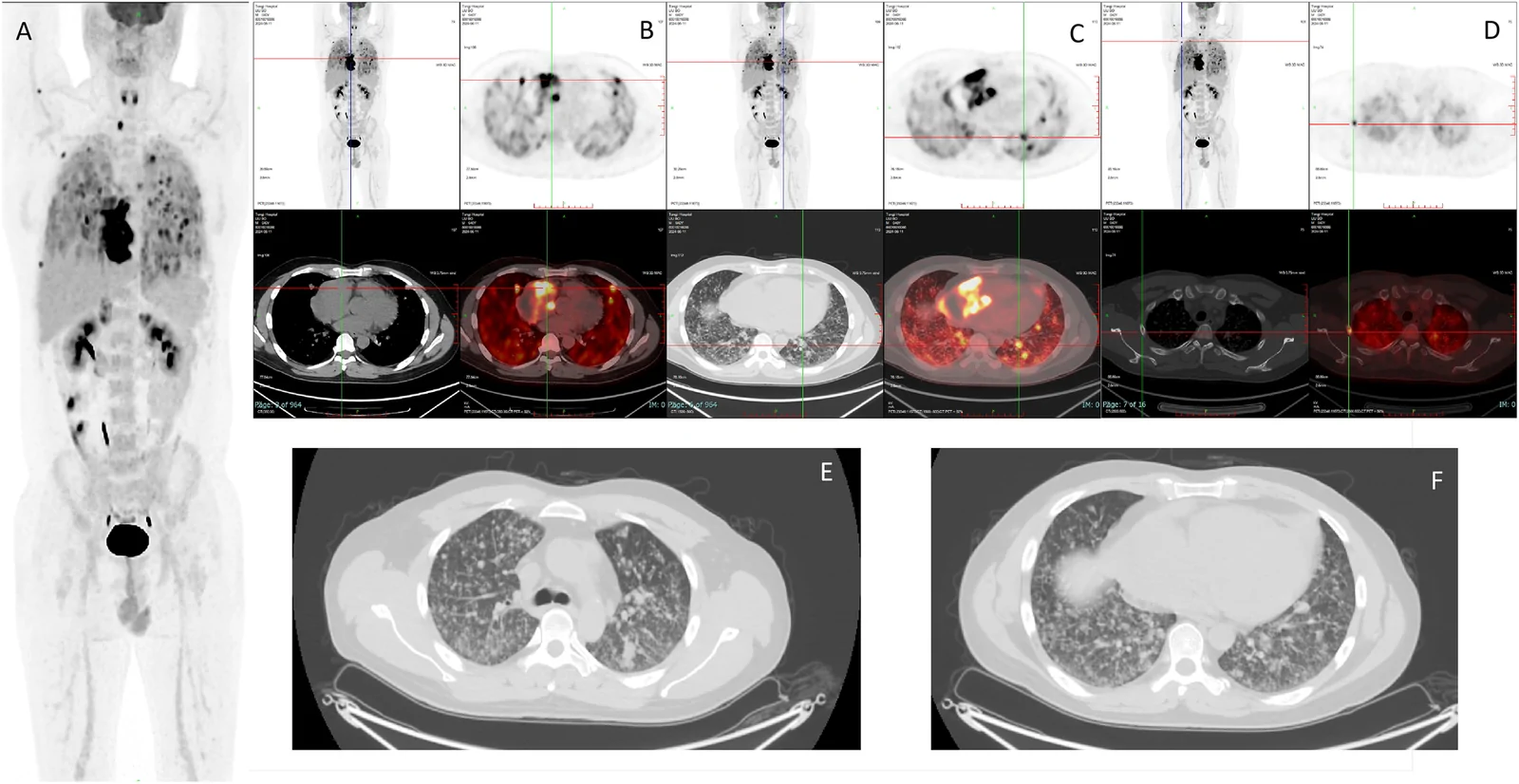
Whole-body PET/CT scan results: **(A–D)** reveal multiple soft-tissue masses/nodules around bilateral atria, pericardium, and mediastinum with elevated metabolism; diffuse lung nodules of varying sizes with increased metabolism; enlarged adrenal nodules with hypermetabolism; and focal metabolic upticks in the right humerus and right third/seventh ribs. **(E,F)** show diffuse lung nodules of different sizes.

A 40-year-old male presented with a 6-month history of a cardiac mass, experiencing sudden syncope without apparent triggers, accompanied by palpitations, chest tightness, fatigue, nausea, vomiting, cough, and expectoration. Transthoracic echocardiography revealed pericardial effusion, a right atrial mass of undetermined nature, and a hypoechoic area posterior to the right atrium, potentially related to the mass. Symptoms partially improved after pericardial blood aspiration, followed by traditional Chinese medicine therapy. One month prior to admission, he developed recurrent cough, hemoptysis, and intermittent low-grade fever, managed with antitussives and antibiotics. Imaging studies demonstrated concerning findings: magnetic resonance imaging (MRI) (7 June 2025) (Figure 1) demonstrated multiple soft-tissue nodules/masses surrounding the bilateral atria and apical pericardium, with right atrial involvement suggestive of neoplasm (mesothelioma suspected), along with mediastinal/epicardial fat proliferation, minimal pericardial effusion, and diffuse pulmonary nodules. Positron emission tomography/computed tomography (PET/CT) (12 June 2025) (Figure 2) further revealed hypermetabolic soft-tissue masses/nodules involving bilateral atria, pericardium, and mediastinum, suspicious for malignancy (sarcoma considered), as well as diffuse hypermetabolic pulmonary nodules, bilateral adrenal nodular thickening, and focal hypermetabolic lesions in the right humerus and right third/seventh ribs, all indicative of metastatic disease.

**Figure 3:**
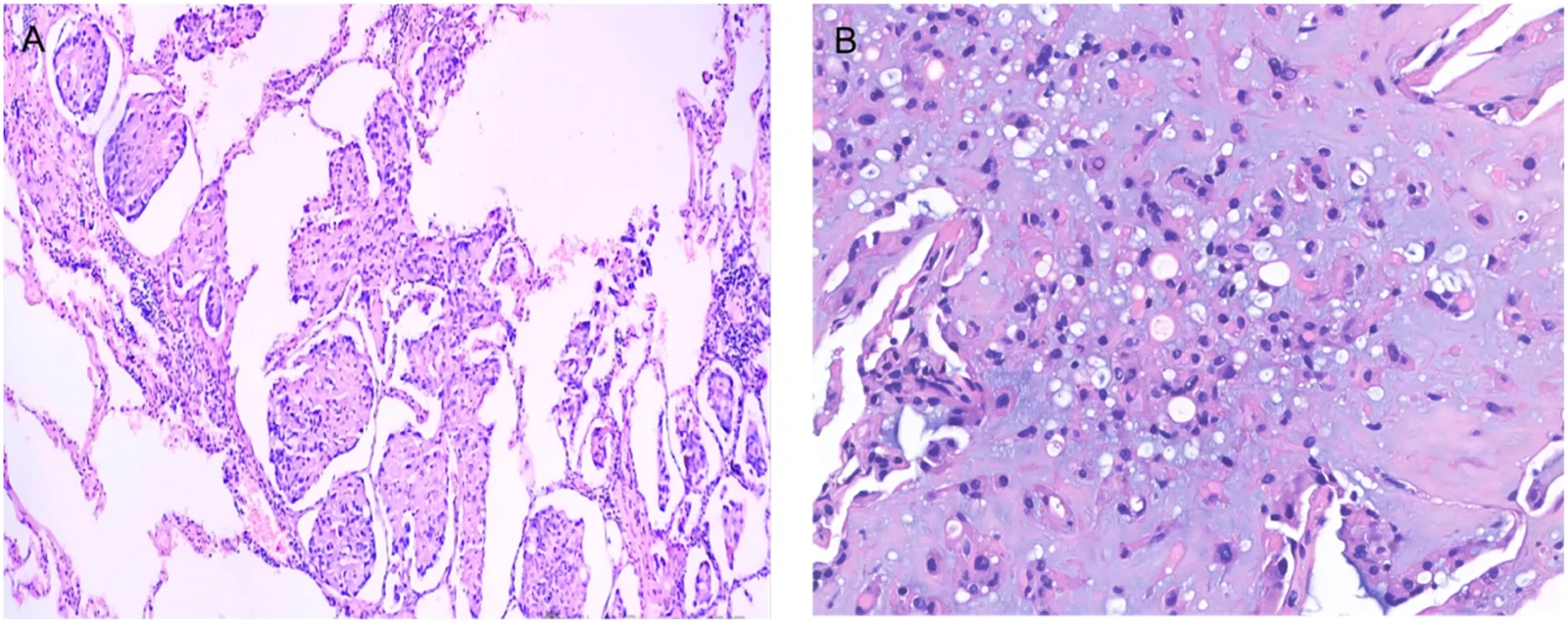
Histological features of the tumor. **(A)** Tumor cells arranged in nests with abundant eosinophilic cytoplasm (×100). **(B)** Intracytoplasmic vacuoles containing erythrocytes (arrows), indicating primitive vascular lumen formation. Tumor cells show low-grade atypia with scattered mitotic figures, filling alveolar spaces in a polypoid pattern while sparing the alveolar septa (×400).

**Figure 4:**
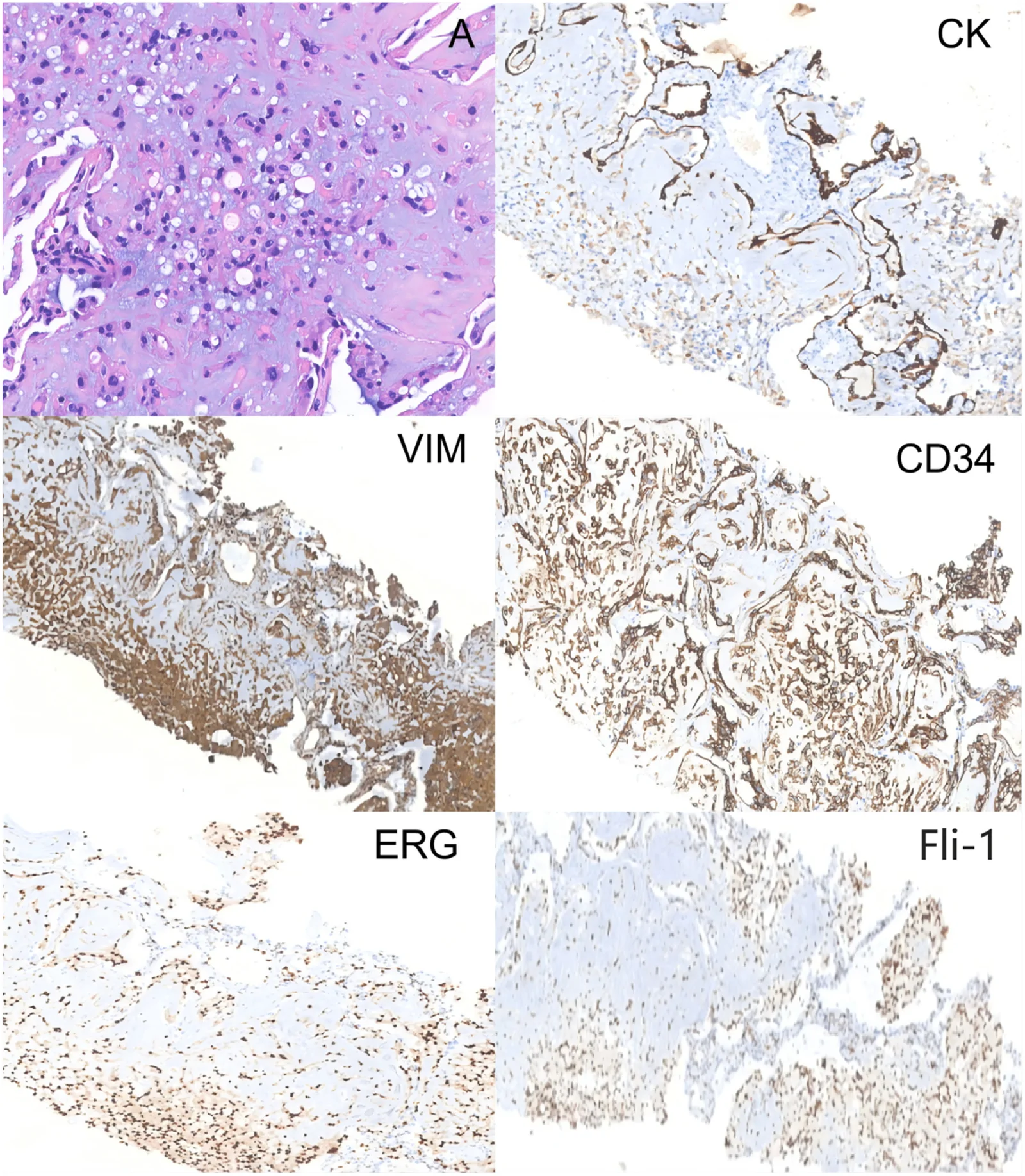
Immunohistochemical findings of the tumor. Representative immunohistochemical staining images showing the immunophenotypic profile of the tumor cells. The tumor cells exhibited diffuse positivity for **VIM**, **CD34**, **ERG**, and **FLI-1**, while showing complete negativity for **CK** (pan-cytokeratin). The absence of CK expression effectively ruled out an epithelial neoplasm (carcinoma), while VIM positivity supported a mesenchymal origin. Importantly, the combined expression of endothelial markers—CD34, ERG, and FLI-1—was consistent with a vascular lineage and strongly supported the diagnosis of EHE (scale bars, 50 μm; original magnification ×200).

On 18 June 2025, the patient underwent video-assisted thoracoscopic pericardial and pulmonary biopsies under general anesthesia. Pathological examination of both specimens revealed identical findings: tumor cells arranged in nests/clusters of varying sizes, with round, epithelioid morphology and abundant eosinophilic cytoplasm. Vacuoles of different sizes containing red blood cells indicated single-cell primitive vascular lumen formation. The cells showed low-grade features with rare mitoses, mild-to-moderate atypia in some, and high density, filling the alveolar spaces in a polypoid pattern while the alveolar septa remained relatively normal (Figure 3). Immunohistochemical (IHC) staining (Figure 4) demonstrated that the tumor cells were positive for VIM, CD31, CD34, ERG, and FLI-1 and negative for PCK (pan-cytokeratin), TTF-1, Napsin A, CK5/6, P63, and Calretinin. The Ki-67 proliferative index was approximately 70%. This immunophenotype—positivity for endothelial markers (CD31, CD34, ERG, and FLI-1) combined with negativity for epithelial and mesothelial markers—supported a vascular origin while effectively excluding carcinoma, mesothelioma, and epithelioid angiosarcoma (the latter typically showing at least focal cytokeratin positivity). The markedly elevated Ki-67 index further argued against an indolent vascular lesion. Collectively, the characteristic histomorphological and immunohistochemical findings were sufficient to establish the diagnosis of EHE.

### Clinical follow-up and management

Surgical resection was precluded by extensive multi-organ involvement (bilateral lungs, pericardium, adrenal glands, ribs, and humerus). After discharge in July 2025, the patient received palliative supportive care with oral Ubenimex (30 mg daily) and regular outpatient surveillance. Long-term telephone follow-up was conducted. At the last telephone follow-up on 7 January 2026—6.7 months post-diagnosis—the patient remained alive without acute deterioration. The therapeutic approach focuses on symptom palliation, quality-of-life maintenance, and tumor growth control, consistent with management principles for aggressive vascular malignancies. Continued surveillance is warranted to monitor for recurrence, metastasis, and adverse events.

## Discussion

Epithelioid hemangioendothelioma (EHE) is a rare, low-grade malignant vascular tumor that can potentially arise in any anatomical location, with the lungs and liver being the two predominant sites. Early studies described pulmonary EHE, formerly termed intravascular bronchioloalveolar tumor (IVBAT), providing fundamental histopathological insights into this entity (1–3, 8). Population-based data further demonstrated its epidemiological features and prognostic profiles (10). Pulmonary EHE (P-EHE) is primarily associated with hepatic and/or osseous EHE (13).

The symptoms of P-EHE are usually non-specific or mild. Distant metastasis of P-EHE is a common occurrence. However, cardiac metastasis has only been reported in cases associated with primary hepatic EHE, while primary cardiac EHE is extremely rare (14). Cardiac tumors can be classified into primary and metastatic types. Among primary cardiac tumors, benign lesions account for 85% and malignant ones for 15%. Compared with primary cardiac tumors, the incidence of secondary (metastatic) cardiac tumors is 20–30 times higher. In the adult population, the most frequently encountered primary cardiac tumors are papillary fibroelastomas and cardiac myxomas, whereas cases of EHE originating primarily in the heart are rarely reported in the literature. Cardiac tumor metastasis can occur through direct spread or indirect pathways such as hematogenous, lymphatic, or intracavitary dissemination (15).

Notably, there have been no reported cases of cardiac EHE with diffuse bilateral pulmonary metastasis. Therefore, in addition to evaluating the lungs and liver, it is crucial to conduct thorough cardiovascular system examinations and assessments during the diagnostic process of EHE to avoid misdiagnosis or missed diagnosis.

PET/CT can reveal increased radiotracer uptake of fluorodeoxyglucose (FDG) in tumors and simultaneously detect EHE involvement outside the lungs, providing valuable reference information for prognosis. In this patient, PET/CT showed increased FDG uptake not only in the bilateral lungs and right atrium but also in the ribs and bilateral adrenal glands. Studies have indicated that an elevated FDG uptake value may be associated with EHE activity and serves as an indicator of poor prognosis (16). Moreover, it provides a reference for determining the suitability of surgical resection. However, due to the partial volume effect, there are significant errors in measuring the standardized uptake values (SUVs) of nodules with a diameter less than 2 cm, which limits their clinical value. Therefore, PET/CT is mainly applied in cases of mass-forming P-EHE. In this patient, high FDG uptake was observed in the lungs, heart, ribs, and adrenal glands, while no uptake was detected in the myocardial region, confirming that the cardiac lesion was solitary and did not involve the myocardium. Combined with MRI examination, it was confirmed that the lesion in the apical region was an abscess formation.

### Diagnostic limitations

We must acknowledge that, in the present case, the diagnosis of EHE was established solely based on histomorphological evaluation (H&E staining) and IHC confirmation. According to the current WHO classification criteria, a definitive diagnosis of EHE requires the demonstration of endothelial differentiation through IHC (e.g., CD31, CD34, ERG, FLI-1) and, ideally, the detection of the characteristic WWTR1-CAMTA1 gene fusion—or its surrogate marker, nuclear TAZ expression—to distinguish it from aggressive mimickers such as epithelioid angiosarcoma, metastatic carcinoma, or epithelioid hemangioma. It is noteworthy that, owing to the patient's financial constraints, further molecular verification could not be performed. We report this case with the aim of emphasizing the necessity for a comprehensive pathological and radiological evaluation in patients with suspected EHE, particularly when the disease presents at atypical anatomical sites.

## Conclusion

In conclusion, cardiac and bilateral pulmonary EHE are extremely rare, with diverse clinical manifestations and a highly variable natural history, making diagnosis challenging. There is a scarcity of basic and clinical research on this condition, necessitating further in-depth exploration. Enhancing awareness of EHE is beneficial for accurate diagnosis and early treatment. When multiple perivascular nodules or masses are present in the lungs or heart, with poor or recurrent responses to symptomatic treatment, and concurrent or sequential lesions occur in the liver, bones, pleura, or other sites, the possibility of EHE should be considered. Early percutaneous biopsy and comprehensive imaging evaluation may facilitate disease diagnosis and improve the patient's prognosis.

## Data Availability

The raw data supporting the conclusions of this article will be made available by the authors without undue reservation.
